# New assembled video laryngoscope: a study on efficacy and cost-effectiveness

**DOI:** 10.1186/cc14282

**Published:** 2015-03-16

**Authors:** SM Ayyan, Z Ali

**Affiliations:** 1 Pariyaram Medical College, Kannur, Kerala, India

## Introduction

Video laryngoscopes have been introduced in recent years as an alternative choice to facilitate tracheal intubation. Difficulties with tracheal intubation are mostly caused by difficult direct laryngoscopy with impaired view to the vocal cords. Many endoscopic intubation laryngoscopes have been designed to visualize the vocal cords around the corner looking through a proximal viewfinder. Although they are useful devices, they have limitations for doing direct laryngoscopy and are very expensive, hence they are not used for routine tracheal intubation.

## Methods

A Macintosh intubating laryngoscope has been modified by attaching a waterproof USB camera with a inbuilt light source, which is located in the same position as the light source on the standard Macintosh blade thus providing a view angle of up to 290° and the USB camera is connected to a laptop. A total of the first 50 patients who presented to the emergency department over a period of 6 months in need of intubation were included in the study and every alternate patient participated in the evaluation of the assembled video laryngoscope (VAL). Information about patient demographics and airway characteristics, Cormack-Lehane (C/L) views and the ease of intubation using the VAL was collected. Failure was defined as more than one attempt at intubation.

## Results

**Figure 1:**
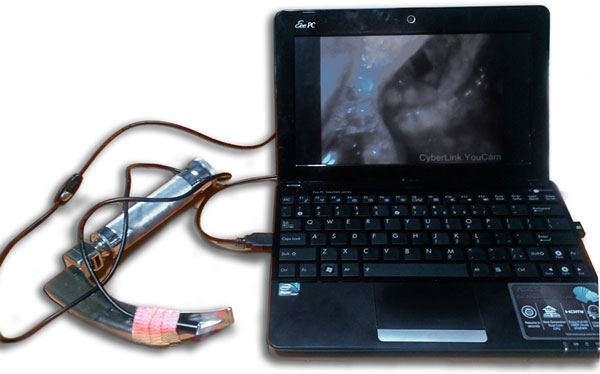
**New video laryngoscope connected to laptop**.

Excellent (C/L1) or good (C/L2) laryngeal exposure was obtained in 92% and 8% of patients respectively. In 25 patients in whom VAL was performed, there was a comparable or superior view. Intubation with direct laryngoscopy was successful in 95.2% of patients and VAL was successful in 95.4% of patients. Three patients from the VAL group and four patients from the direct laryngoscopy group were excluded. See Figure 1.

## Conclusion

This new assembled VAL is the cheapest video-assisted laryngoscope available costing around $60, which can even be introduced into primary healthcare setup in developing countries. VAL consistently yielded a comparable or superior glottic view compared with direct laryngoscopy despite the limited or lack of prior experience with the device. Because the device can be used for both routine as well difficult tracheal intubation, it may be a helpful tool to intubate trauma cases where C-spine immobilization is unavoidable. The presented video-assisted laryngoscope is a useful tool for documentation, teaching and monitoring tracheal intubation.

